# Truncated Rep protein of porcine circovirus 2 (PCV2) caused by a naturally occurring mutation reduced virus replication in PK15 cells

**DOI:** 10.1186/s12917-019-1984-8

**Published:** 2019-07-15

**Authors:** Yi Hu, Xiong Cai, Yang Zhan, Xiaomin Yuan, Tanbin Liu, Lei Tan, Yalan Li, Lijie Zhang, Lingchen Yang, Wei Liu, Zhibang Deng, Naidong Wang, Yi Yang, Shiyin Guo, Aibing Wang

**Affiliations:** 1 Lab of Animal Models and Functional Genomics (LAMFG), The Key Laboratory of Animal Vaccine & Protein Engineering, College of Veterinary Medicine, Hunan Agricultural University (HUNAU), Changsha, 410128 Hunan China; 2 Institute of Innovation and Applied Research in Chinese Medicine, Hunan University of Chinese Medicine, Changsha, 410208 China; 3 Lab of Functional Proteomics (LFP), The Key Laboratory of Animal Vaccine & Protein Engineering, College of Veterinary Medicine, HUNAU, Changsha, 410128 Hunan China; 4 State Key Laboratory of Medicinal Chemical Biology and College of Pharmacy, Nankai University, Tianjin, 300071 China; 5 College of Food Science and Technology, HUNAU, Changsha, 410128 Hunan China

**Keywords:** Porcine circovirus 2(PCV2), Truncated Rep protein, Infectious clone, Rescued viruses, Virus replication, PCV2 evolution

## Abstract

**Background:**

Porcine circovirus 2 (PCV2) is the causative agent of porcine circovirus-associated diseases (PCVADs). The infection of PCV2 is widespread and has serious consequence, thereby causing significant economic losses in the swine industry worldwide. Previously, we found that a strain named YiY-3-2-3 has a naturally occurring point mutation (G^710^ to A^710^) in ORF1 region, which leads to a shorten product of the *rep* gene (945 to 660 base pair). Importantly, the Rep protein is responsible for genome replication of PCV2. To explore the effects of this mutation on the PCV2 replication, in the current study we constructed infectious clone of this IF-YiY-3-2-3, as well as those of its two parental strains of IF-YiY-3-2-1 and IF-YiY-3-2-10. Subsequently, these infectious clones which have 1.1 copy of PCV2 genome of their corresponding strains were transfected into PK15 cells to obtain rescued viruses, respectively.

**Results:**

Though all of the three infectious clones could be rescued, the copy number and infectivity of these rescued viruses were significantly different, as analyzed by fluorescence quantitative PCR, Tissue culture infectious dose 50 (TCID_50_), and indirect immunofluorescence assay (IFA). Notably, whether the PCV2 copy number, viral titer or the infectivity of rescued viruses from infectious clone IF-YiY-3-2-3 was significantly less than those of its parental clones. Meanwhile, the spatial structure of the Rep protein from the IF-YiY-3-2-3 displayed an apparent truncation at the C-terminal.

**Conclusions:**

These findings therefore suggest that the Rep protein with truncated C-terminal would reduce virus replication and infectivity, and there might also exist both favorable and unfavorable mutations in the ORF1 of PCV2 in the process of its evolution.

## Background

Porcine circovirus 2 (PCV2), belonging to the genus Circovirus of the family Circoviridae, is a small, circular, single-stranded DNA virus [1]. PCV2 causes the intumescentia of lymph node, weight loss, jaundice, anemia and diarrhea mainly in 10–15 weeks old piglets, thus resulting in low productivity and enormous economic losses [2, 3]. PCV2 is considered the major etiological agent of porcine circovirus associated diseases (PCVADs), which includes postweaning multisystemic wasting syndrome (PMWS), porcine dermatitis and nephropathy syndrome (PDNS), porcine respiratory disease complex (PRDC), congenital tremors type A-II (CT), and reproductive failure [4–6].

The PCV2 genome is 1767 or 1768 nucleotides (nt) in length and contains three major open reading frames (ORFs), namely, ORF1, ORF2 and ORF3. ORF1 encodes two replication-related proteins, Rep and Rep’; ORF2 encodes the major structural capsid (Cap) protein which is also the major immunogenic protein; ORF3 encodes an apoptosis-inducing/pro-apoptotic protein [7–9]. Currently, PCV2 is classified into five major groups: PCV2a, PCV2b, PCV2c, PCV2d and PCV2e, and the genetic distance between these different genotypes is at least 0.035 [10, 11]. Additionally, PCV2a and PCV2b can be further divided into five subgroups (2A-2E) and two subgroups (1A-1B), respectively. The occurrence of high genetic variation and genotype shift often leads to the prevalence of a major genotype or the coexistence of different genotypes of PCV2 in diverse regions and different periods [12, 13]. Nowadays, PCV2b and PCV2d are deemed to be the predominantly circulating genotypes, and increase the pathogenicity of PCVAD [12, 14–17].

Accumulating studies have confirmed that PCV2 is continuously evolving through genome recombination or point mutation [18, 19]. Indeed, the manner of point mutation for PCV2 evolution has been described by many studies [20–23]. In this regard, we, in a previous study, not only confirmed the individual animal or the individual organ from this animal co-infected with diverse PCV2 genotypes, or even sub-genotypes, but also provided evidence of diverse point mutations acquired during immune escape by PCV2. Additionally, we noticed that the occurrence of point mutations exists in both ORF1 and ORF2 of PCV2 [24], as consistent with previous reports [25, 26]. However, the biological importance and significance of novel amino acid alterations, especially those in ORF1, are less characterized and explored.

In this study, we intended to investigate the consequence of one naturally occurring mutation (G^710^ to A^710^) in the ORF1 region of a recently reported PCV2 strain (YiY-3-2-3), which potentially has a shortened Rep protein [24]. To this aim, we compared the replication capability of this PCV2 mutant strain with its two parental strains YiY-3-2-1 (PCV2b-1B) and YiY-3-2-10 (PCV2d) [24] by constructing the infectious clones of them. Meanwhile, we analyzed the infectivity of these recovered viruses through a series of methodologies.

## Methods

### Samples collection and PCV2 detection

The collection of clinical samples (spleen, lymph node, and lung) and the preparation of genomic DNAs have been described previously. The presence of PCV2 was identified by PCR using a forward primer ZY009 and a reverse primer ZY010 (Table 1) [24]. PCR amplification was initiated by preheating at 94 °C for 5 min, followed by 30 cycles at 94 °C for 30 s, 60 °C for 30 s, and 72 °C for 45 s, and a final extension at 72 °C for 10 min. PCR products were analyzed on 1% agarose gel electrophoresis.

**Table 1:** Primers used in this study

Primer	Sequence (5′-3′)	Size
ZY009	CCATATGAAATAAATTACTGAG	785 bp
ZY010	CAGCGCACTTCTTTCGTTTTCAG	
ZY001	CGGGGTACCACTGAGTCTTTTTTATCACTTCG	1777 bp
ZY002	CCCAAGCTTAAGACTCAGTAATTTATTTCATATGG	
HY008	CGGGGTACCTCCTTGGATACGTCATATCTGAAAACG	1077 bp
HY009	CCATTACGAAGTGATAAAAAAGACTCAGTAATTTATTTCATATGG	
HY010	GAAATAAATTACTGAGTCTTTTTTATCACTTCGTAATGG	862 bp
HY011	CCCAAGCTTTCTTTTTGCTGGGCATGTTGCTGC	
HY012	CGGGGTACCTCCTTGGATACGTCATCGCTGAAAACG	1077 bp
HY013	CCCAAGCTTTCTTCTTGCTGGGCATGTTGCTGC	
ZY097	GGGTTATGGTATGGCGGGAG	192 bp
ZY098	CCCTCACTGTGCCCTTTGAA	

Note: The underlined sequences represent restriction enzyme sites introduced

### PCV2 genomic DNA amplification and sequencing

The complete genome of PCV2 was amplified from the sample DNAs using a set of primers designed according to the sequence of a PCV2 strain (GenBank No. AY321991). The forward primer was ZY001 and the reverse primer was ZY002 (Table 1). PCR amplification was initiated by preheating at 94 °C for 5 min, followed by 25 cycles at 94 °C for 30 s, 60 °C for 30 s, and 72 °C for 60 s, and a final extension at 72 °C for 10 min. PCR products were analyzed on 1% agarose gel electrophoresis, followed by extraction with a DNA Gel Extraction Kit (omega, USA). The purified PCR products were cloned into pSP-72 cloning vector (Promega, Madison, WI, USA) and sequenced by genscript (Nanjing, China). The whole genome sequence was assembled by using DNAstar.

### Construction of infectious clones

**Fig. 1:**
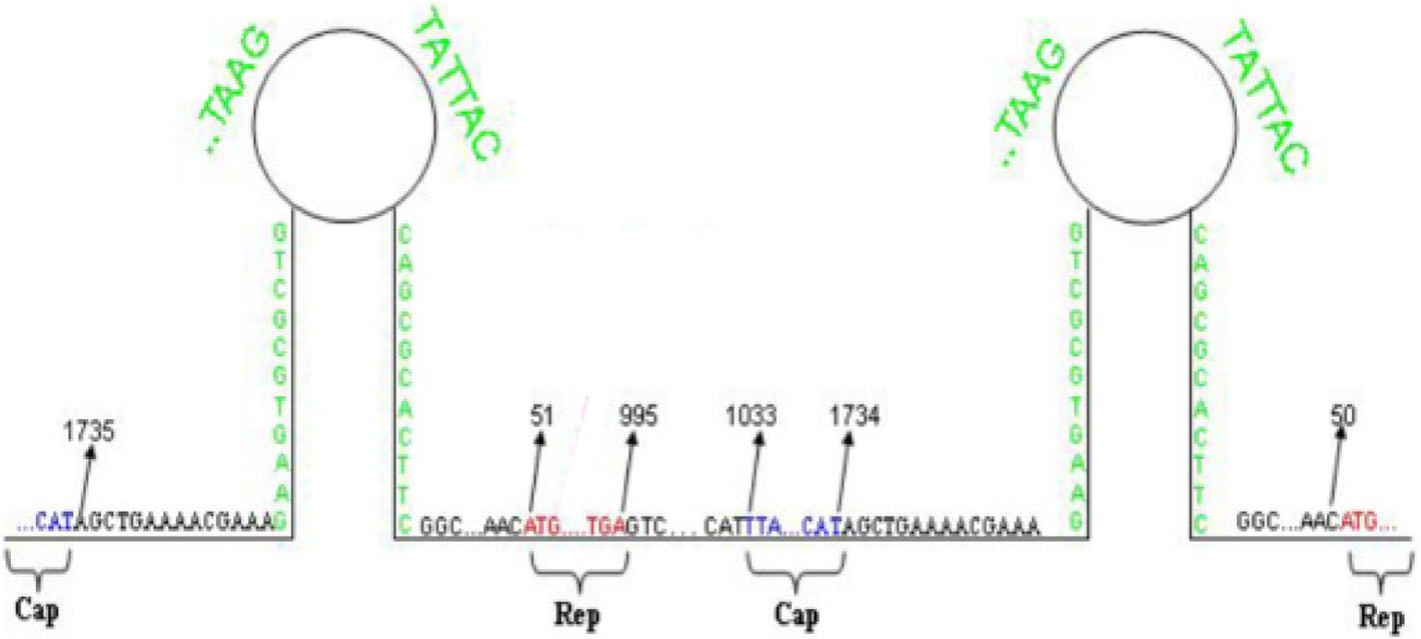
Diagram demonstrating the constructed infectious clone which includes a 1.1 copy of PCV2 genome, additionally, the location of the major ORFs encoding structural and non-structural proteins are also indicated

To construct the infectious clones, a set of primers including HY008, HY009, HY010, HY011 for pSP72-PCV2b-1B-YiY-3-2-1 (IF-YiY-3-2-1) and pSP72-PCV2b-1B-YiY-3-2-3 (IF-YiY-3-2-3), a set of primers containing HY010, HY011, HY012, HY013 for pSP72-PCV2d-YiY-3-2-10 (IF-YiY-3-2-10), were designed and synthesized to amplify the DNA fragment of 1.1 copy PCV2 genome via PCR (Table 1 and Fig. 1) [27]. PCR products were separated on 1% agarose gels by electrophoresis. DNA band having full length PCV2 genome was excised and purified by using a kit (Omega, USA). The plasmids of infectious clones were constructed by inserting the 1.1 copy of PCV2 genomic DNA fragment into double-digested pSP72 vector (*Kpn*I and *Hind*III), respectively. The resultant plasmids named IF-YiY-3-2-1, IF-YiY-3-2-3 and IF-YiY-3-2-10 were verified by restriction enzyme *Kpn*I and *Hind*III digestion and DNA sequencing.

### Transfection of infectious clone plasmids

Constructed infectious clones were separately transfected into PK15 cells seeded into 6-well plate using Lipofectamine 2000 according to the manufacturer’s instructions. Normal PK15 cells served as negative control. The cells were incubated for 8 h at 37 °C with 5% CO_2_ in a serum-free DMEM, then discarded the media and incubated with fresh DMEM containing 5% FBS (fetal bovine serum) for serial passages. The rescued viruses cultured via serial passages was separately collected from the cell supernatants and stored at − 20 °C.

### Transmission Electron microscopy (TEM)

Seventy-two hours after the transfection of the infectious clone plasmids into PK15 cells, the media and cells were collected, followed by three freeze-thawed cycles, and then the cell debris were removed by centrifugation [28]. The morphology of the rescued viruses, together with that of the PCV2 virus-like particle (VLP) described before [29], were examined by TEM.

### Indirect immunofluorescence assay (IFA)

PCV2-free PK-15 cells were grown in 48-well plate until reaching 70–80% confluency. Each plate was inoculated with identical amounts of various rescued viruses (10^8^ copy number) or the positive control rescued viruses which were produced by transfecting PK15 cells with the infectious clone of PCV2d (Genbank number: KP112486), respectively. After 2 h of incubation, the inoculum was removed and washed three times with PBS. DMEM medium (Gibco BRL Life Technologies, NY, USA) containing 5% FBS was added and the infected cells were incubated at 37 °C for 48 h. Indirect immunofluorescence assay (IFA) was performed to detect infectious viruses. Rabbit anti-PCV2 serum (1:1000) diluted with 1% BSA was added to the wells, then incubated with a fluorescein isothiocyanate (FITC)-conjugated anti-rabbit immunoglobulin donkey IgG (1:1000) (Gibco BRL Life Technologies, NY, USA). The fluorescent signals were observed by a fluorescent microscope (Olympus, Tokyo, Japan). For the quantification of the percentage of cells infected by PCV2 viruses, cells in at least five random fields were examined and calculated. All assays were repeated at least three times.

### Real-time PCR analysis

Real-time PCR primers were designed by primer-blast according to the virus-specific sequence (Genbank number: KJ867555). Primer pairs ZY097 and ZY098 (Table 1) were used for real-time PCR to calculate the virus load in the supernatants of 1st to 6th passages. The real-time PCR assay was conducted with SYBR Green Master Mix (Vazyme, China) and 2 μl of virus in 20 μl reaction mixtures. The parameter for real-time PCR consisted of 40 cycles of denaturation at 95 °C for 10 s, annealing and extension at 60 °C for 30 s. All assays were repeated at least three times with each experiment performed in triplicate.

### Tissue culture infectious dose 50 (TCID_50_) assay

TCID_50_ assay was used to determine the titers of the rescued viruses. Briefly, the rescued viruses of 3rd to 6th passages were 10-fold serially diluted in DMEM medium supplemented with 2% FBS and antibiotics. Each rescued virus dilution was inoculated into four separate wells containing about 2 × 10^4^ PK15 cells. After absorption for 2 h at 37 °C, the liquids in the wells were removed, and DMEM with 2% FBS was added to the wells. Plates were incubated for an additional 72 h at 37 °C. After the media were removed, the cells were fixed with 4% paraformaldehyde-PBS solution for 20 min at room temperature and washed three times with PBS. Positive cells were made visible through the IFA method using anti-PCV2 antibody as described above. Virus titers in cell cultures for each passage were then determined by a microtitration infectivity assay and recorded as TCID_50_/ml by using the Reed-Muench method [30]. The TCID_50_ assay was at least repeated three times.

### One-step growth curve experiment

One-step growth curve assay was particularly performed to determine the growth of the rescued viruses (including the Positive control, YiY-3-2-3, YiY-3-2-1 and YiY-3-2-10) [31]. Briefly, PCV2-free PK-15 cells were grown in a 12-well plate until the density reaching 50%. Each well was inoculated with identical amounts of rescued viruses. Both the supernatant and cell pellet were collected at different time intervals after inoculation. One-step growth curve was drawn according to the results of fluorescence quantitative PCR. The assay was at least repeated three times.

### Three-dimensional mapping of amino acids in the Rep protein of PCV2

The Rep amino acid residues among the three PCV2 isolates were aligned through the website (http://multalin.toulouse.inra.fr/multalin/). The spatial structures of these three PCV2 Rep proteins were analyzed by mapping them with a solid surface, simulated with the modeller software. Three-dimensional structures of the PCV2 Rep protein were displayed with PyMOL version 1.7.4.4 (www.pymol.org) [32].

### Statistical analysis

All assays were repeated at least three times, with each experiment performed in triplicate. One-way ANOVA was used to compare results between different groups. All statistical analysis was performed using SPSS version 16.0. *P < 0.05, **P < 0.01, ***P < 0.001, NS, non-significant.

## Results

### The generation of rescued viruses for mutant PCV2 and its parental strains

The YiY-3-2-3 strain had a G^710^ to A^710^ point mutation in the ORF1 region, which leads to a shortened *rep* gene (from 945 to 660 base pairs) in length, thereby encoding a carboxyl-terminally truncated Rep protein with 219 amino acids (aa) instead of 315aa in length. To investigate the effect of this G^710^ to A^710^ mutation in ORF1 on PCV2 virus replication, we constructed the infectious clones of this mutant strain and its two parental strains. These three infectious clone plasmids which respectively contain 1.1 copy of PCV2 genome illustrated in Fig. 1 were confirmed by the digestion of double restrictive enzymes since an appropriate 1.9 kb inserted fragment could be observed. Additionally, DNA sequencing also verified the sequences. Successfully constructed infectious clone plasmids were designated as IF-YiY-3-2-1, IF-YiY-3-2-3 and IF-YiY-3-2-10, respectively, and transfected into PK15 cells for the generation of rescued viruses.

### The morphology and infectivity of rescued viruses

**Fig. 2:**
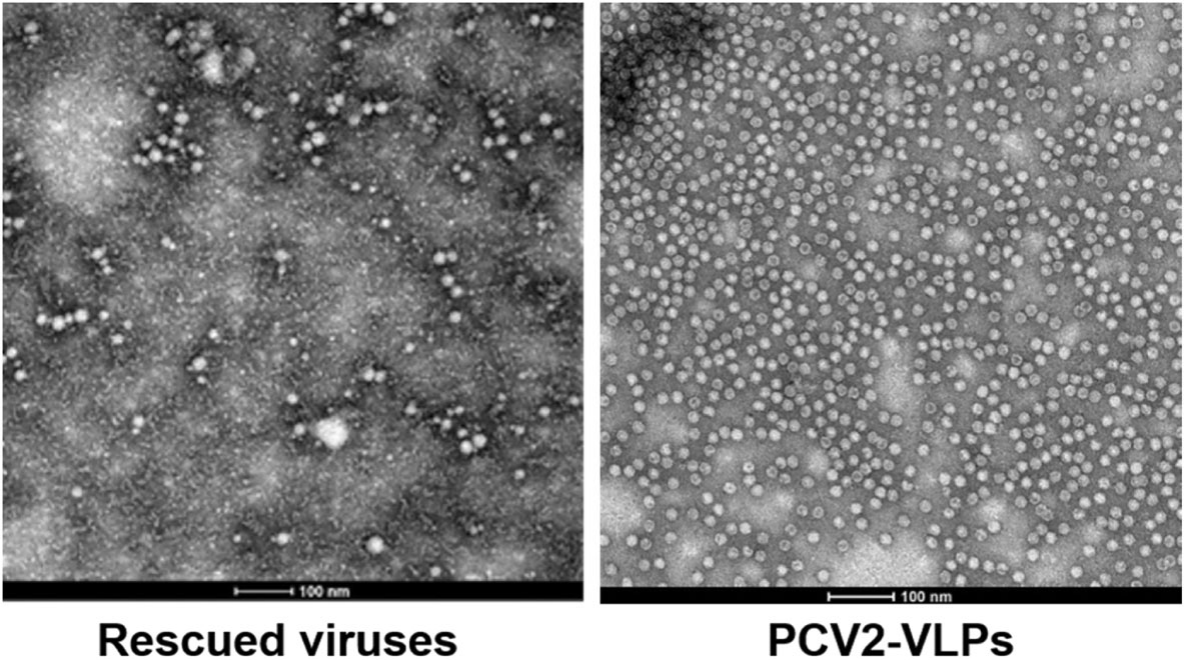
The morphology and microstructure of the rescued viruses produced from PK15 cells, the representative image indicated that the rescued viruses have a spherical morphology with a size of 17–22 nm in diameter, as similar to those of PCV2-VLPs

**Fig. 3:**
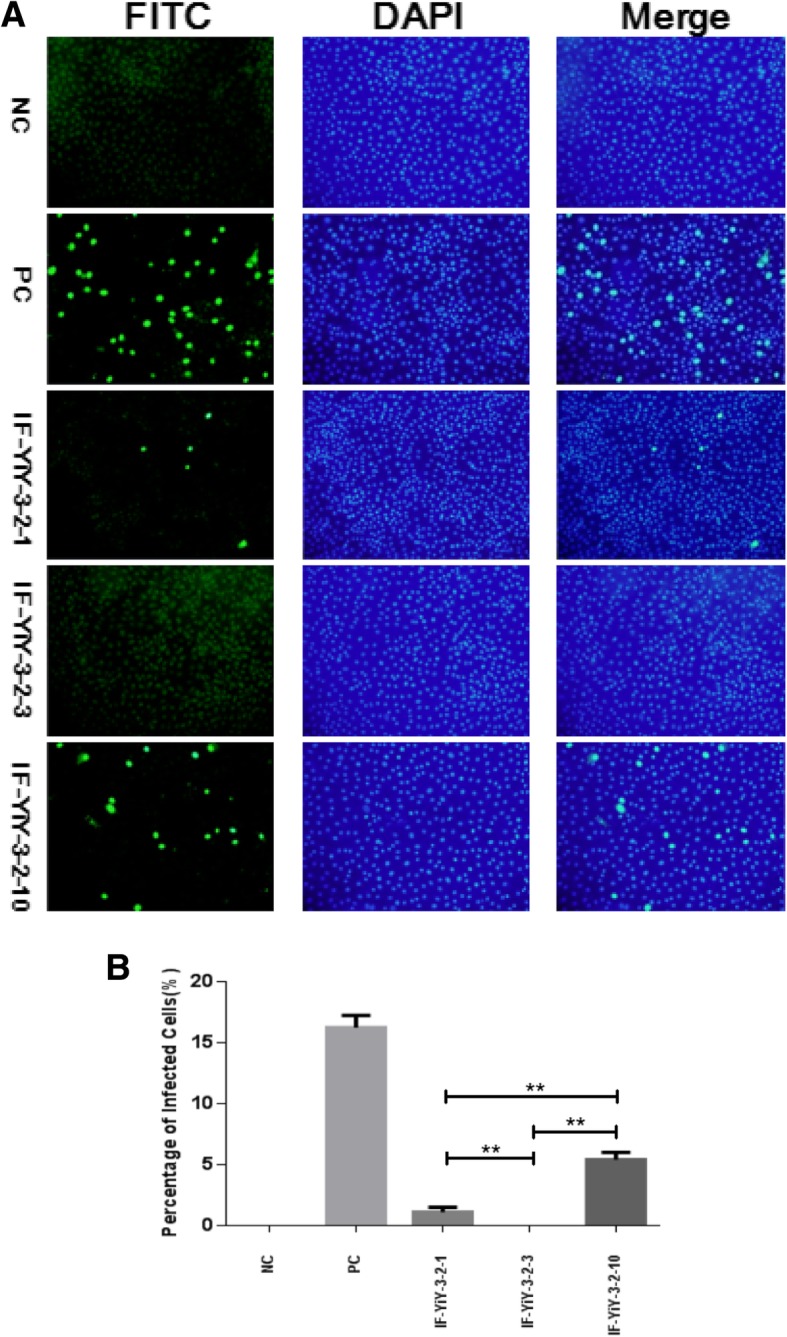
IFA results of PK cells infected by PCV2 rescued viruses. **a** Shown were representative IFA images (Magnification: 20 × 10, Olympus), NC, negative control; PC, positive control. **b** Quantification of PK15 cells infected by PCV2 viruses (*n* = 5). Results were expressed as mean ± SD. IFA assays were independently repeated three times. **represents statistical significance *P*<0.01 between indicated groups

The rescued viruses were firstly examined by TEM. As shown in Fig. 2, the viruses had a spherical morphology with a size of 17–22 nm in diameter, as similar to those of PCV2-VLPs, suggesting that the viruses derived from infectious clones were successfully rescued. To substantiate this conclusion, we performed IFA to analyze the infectivity of these viruses. IFA results reflected an important fact that the rescued viruses had the ability to infect PK15 cells and possessed good reactivity and specificity against the anti-PCV2 antibody, while the infectivity of them was significantly different (Fig. 3a). The quantification of PCV2-positive cells demonstrated that the wild type or positive control viruses had the highest infectivity (16.25%), followed by the rescued viruses from IF-YiY-3-2-10 and IF-YiY-3-2-1 infectious clones, whereas no or few fluorescent signals were observed in IF-YiY-3-2-3 rescued viruses infected cells, as almost comparable to those in uninfected cells. The statistical analysis clearly demonstrated significant differences among these three rescued viruses (*P*<0.01) (Fig. 3b).

### The analysis of viral load and titer

**Fig. 4:**
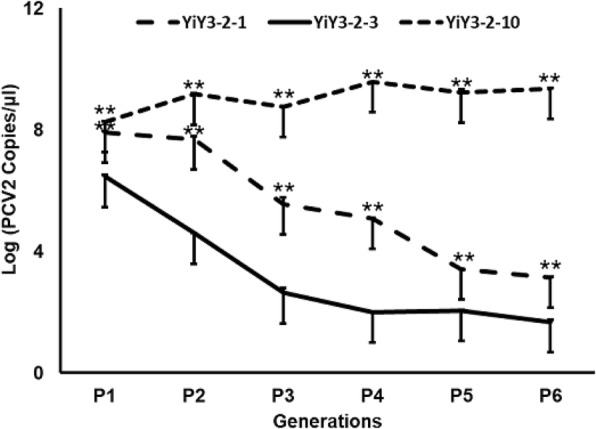
The virus load of PCV2 from 1st to 6th generation of serial passages was reflected by determining PCV2 copy number using quantitative real-time PCR. The X-axis shows the generations examined, while the Y-axis indicates the viral load as the logarithm of the copy number in the cell supernatants. Results were expressed as mean ± SD. Real-time PCR was repeated at least three times with each experiment performed in triplicate. **represents statistical significance *P*<0.01 among different groups

To further analyze the effect of G^710^ to A^710^ point mutation in the ORF1 on PCV2 virus replication, the rescued viruses from passage 1st to 6th (e.g. 1–6 generation) were respectively harvested from PK15 cells, and subjected to quantitative real-time PCR assay for the measurement of PCV2 DNA copy number. As indicated in Fig. 4, the viral load in the supernatants of those three rescued viruses displayed pronounced differences among them (*P*<0.01). Several characteristics could be summarized: a) the viral load from rescued viruses YiY-3-2-1 and YiY-3-2-10 was similar at the beginning (namely, 1st generation), a slight increase in viral load was observed for YiY-3-2-10, while a markedly decrease in viral load was seen for YiY-3-2-1 with the increase of passages; b) the initial viral load of YiY-3-2-3 was lower than those of the other two, furthermore, the downward trend of the viral load from 1st to 6th generation was more pronounced than that of YiY-3-2-1; c) from 1st to 6th generation the viral load for those three rescued viruses demonstrated an order of YiY-3-2-10 > YiY-3-2-1 > YiY-3-2-3.

**Fig. 5:**
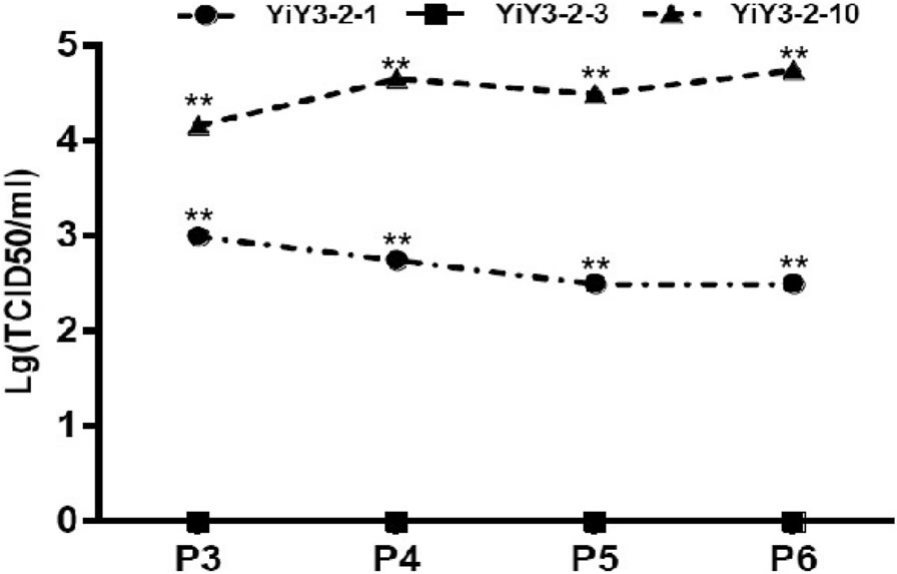
The viral titers of PCV2 from 3rd to 6th generation of serial passages was determined by IFA assay. The X-axis indicates the generations tested, while the Y-axis shows the viral titer as the logarithm of the TCID_50_ in the cell supernatants. **represents statistical significance *P<*0.01 among different groups

Additionally, the viral titer of rescued viruses from passage 3rd to 6th (e.g. 3–6 generation) was also determined by IFA [30]. As indicated in Fig. 5, the viral titer of those three rescued viruses displayed significant differences among them (*P*<0.01). The results were generally in accordance with the data of the detection of PCV2 genomic copy number. Notably, the titers for YiY-3-2-3 rescued viruses were almost undetected in the whole process, and the TCID_50_ for the P1 and P2 generations of YiY-3-2-10 and YiY-3-2-1 remained low level and non-significant difference (Data not shown).

### The virus growth of the mutant strain YiY-3-2-3

**Fig. 6:**
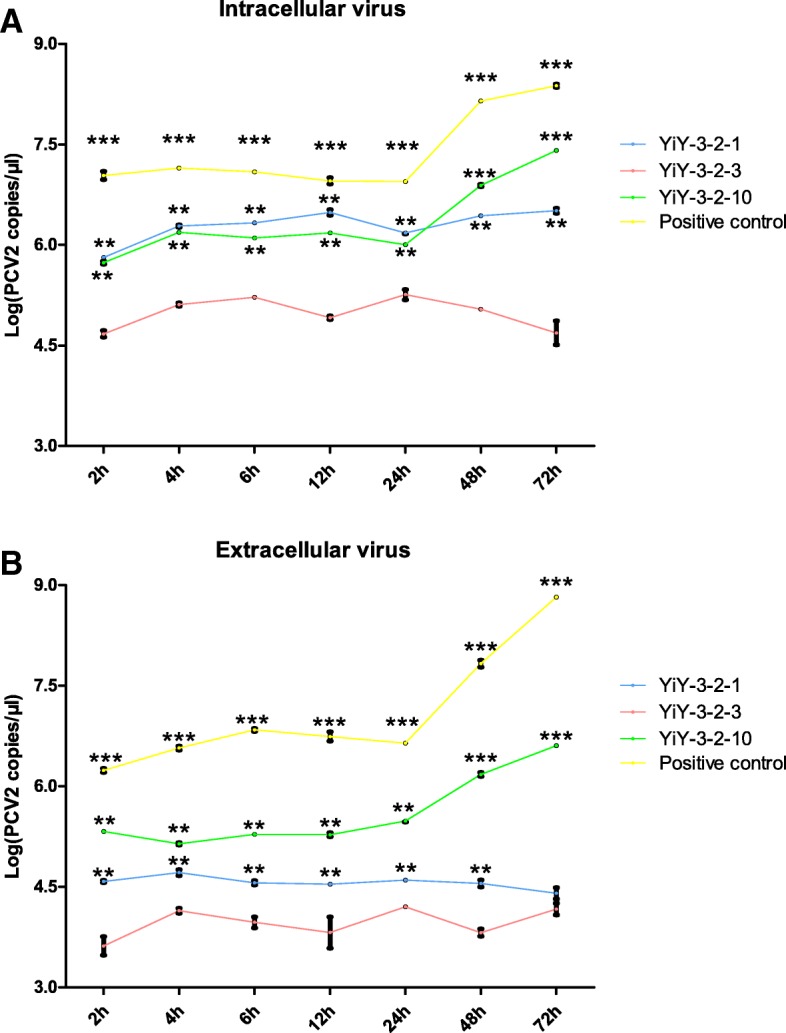
The one-step growth curves of the rescued viruses (including the Positive control, YiY-3-2-3, YiY-3-2-1 and YiY-3-2-10) were performed by determining PCV2 genomic copy number using quantitative real-time PCR. **a** The content of the intracellular virus; **b** The content of the extracellular virus. The X-axis shows the incubation time, while the Y-axis indicates the viral load as the logarithm of the copy number of intracellular and extracellular virus. Results were expressed as mean ± SD. This assay was repeated three times. ** and *** represents statistical significance *P*<0.01 and *P*<0.001 relative to the YiY-3-2-3 group, respectively

To explore whether the strain YiY-3-2-3 losses its ability to replicate, a one-step growth curve experiment was performed to determine the virus contents in both the supernatant and the cell pellet. As indicated in Fig. 6, the results for the contents of intracellular and extracellular viruses demonstrated that following the infection of PK15 cells with YiY-3-2-3 rescued viruses, there were no significant changes in the virus contents within 72 h, which are significantly different from the one-step growth curves of the positive control and YiY-3-2-10 [31].

### The aa sequence and structural analysis for the Rep proteins of the mutant and parental strains

**Fig. 7:**
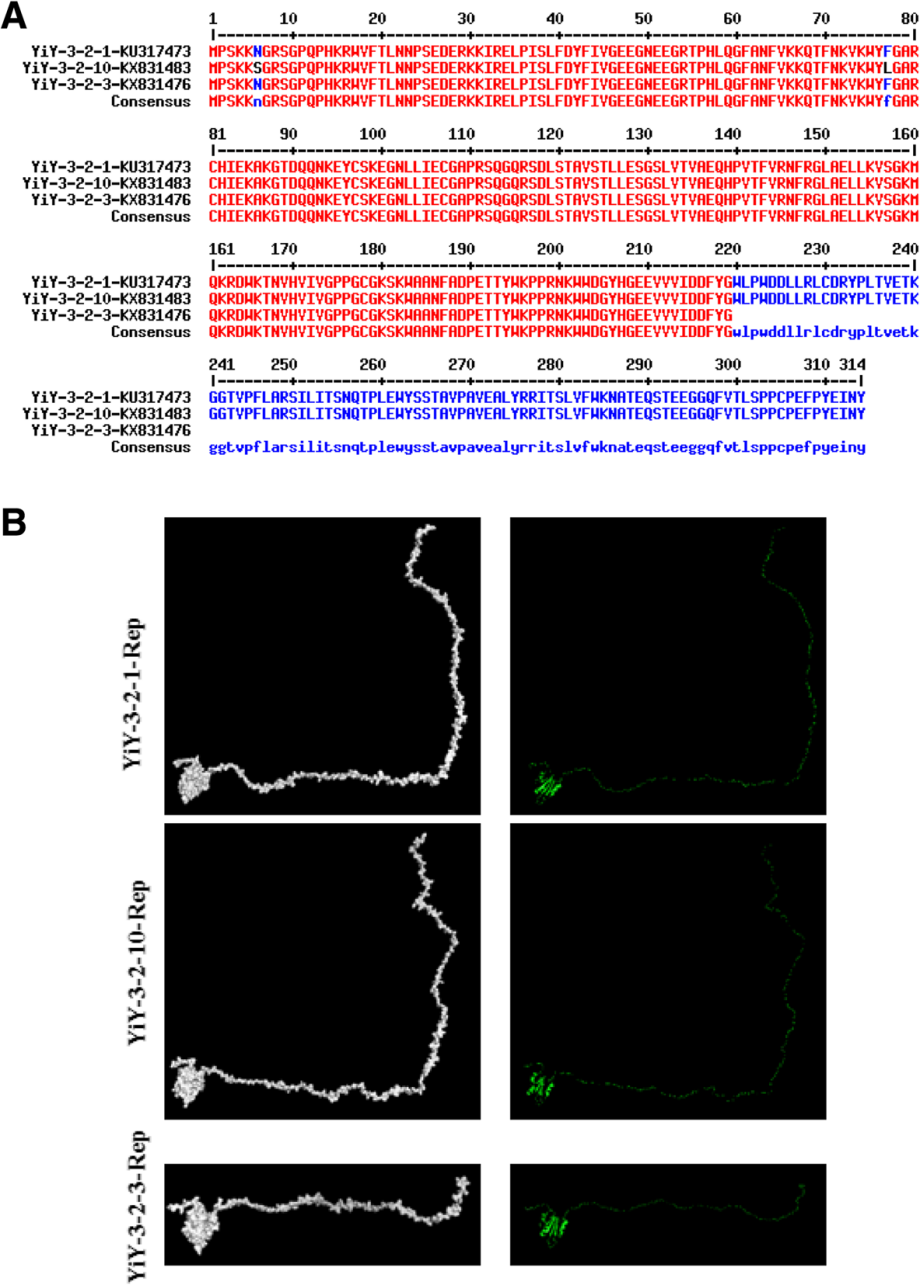
Primary amino acid sequence alignment and three-dimensional mapping of amino acids for the three PCV2 Rep proteins. **a** Comparison of the sequences of amino acid residues in the Rep proteins among the three PCV2 isolates. (GenBank accession numbers KU317473, KX831483 and KX831476). **b** The surface structures of PCV2 Rep proteins were generated based on the amino acid sequences, and displayed using PyMOL

To study the effect of single amino acid alteration on the structure of PCV2 Rep protein, primary amino acid sequence alignment of the three PCV2 Rep proteins from the mutant YiY-3-2-3 and its parental strains were conducted by using MultAlin, and the spatial structures of them were simulated by mapping with a solid surface. As shown in Fig. 7a, there were two amino acid alterations in the ORF1 of the two parental strains, while the Rep protein from the mutant YiY-3-2-3 strain only had 219 amino acids, 95 amino acids less than those of the other two parental strains. Consequently, the spatial structure of the Rep protein from theYiY-3-2-3 strain had an apparent truncation at the C-terminal, while those of the two parental strains were highly similar.

## Discussion

The genome of PCV2, being in a closed stem-loop shape (Fig. 1), is replicated in a rolling circle manner [33], early studies constructed infectious clone of it through the ligation of two copies of PCV2 genomic DNA, thereby facilitating to enhance the replication feasibility and form continuous genome for progeny viruses [34]. In this study we used 1.1 copy of PCV2 genome including two in tandem PCV2 origins to generate infectious clones, as consistent with a recent report [35]. Importantly, rescued viruses were obtained and confirmed, as indicated by TEM observation and IFA assay. Therefore, our work and previous reports collectively suggest that a length of about 1.1 copy of PCV2 genome may represent the minimum requirement for successfully constructing PCV2 infectious clone and obtaining rescued virus.

As mentioned above, the major immunogenic capsid (Cap) protein encoded by the ORF2 of PCV2 is tightly related to the pathogenicity of this virus, the mutations in the ORF2 therefore are much concerned and intensively studied [30, 36–39]. In contrast, the mutations occurred in the ORF1 are rarely reported, mainly because the Rep protein and the splice variant Rep*’* protein are regarded just responsible for PCV2 genome replication [40]. However, several studies have demonstrated that things may be not so simple. For instance, PCV1 has 86% amino acid sequence identity and antigenic cross-reactivity with PCV2, while it is non-pathogenic. Furthermore, a chimera incorporating ORF1 from PCV1 and ORF2 from PCV2 is also non-pathogenic in pigs [41]. These suggest that ORF1 not only is responsible for encoding Rep and Rep*’* proteins but also contributes to the alteration in the pathogenicity of PCV2. So far, there was only one report describing the effects of the mutations in the ORF1 on PCV2 replication, while these mutations reported were artificially created [42]. Thus, our study should be the first report characterizing the effect of naturally occurring mutation in the ORF1 on the PCV2 genome replication. More importantly, the results of IFA, viral load assay and viral titration commonly indicated that the G^710^ to A^710^ mutation in the ORF1 causing a shortened Rep protein significantly reduced virus replication and infectivity. In accordance with this, the results of the simulation of non-homologous structure also confirmed that the Rep protein of this mutant PCV2 strain is truncated, while those of its two parental strains remain highly similar. These results suggest that single amino acid change may be not enough to affect the major structure of Rep protein unless this alteration leads to a completely structural abnormality (e.g. a truncated Rep protein reported here). This may at least partially explain the differences observed in virus replication and infectivity among these PCV2 strains, though the structures modeling of PCV2 Rep protein are not so ideal due to lacking of a reference. Therefore, this finding not only strengthened the contribution of the ORF1 to PCV2-associated pathogenesis, but also provided the first and direct evidence that in the process of PCV2 evolution there may also exist both favorable and unfavorable mutations in the ORF1, implying the possibility to find higher titer viruses for producing more effectively inactivated vaccine to prevent porcine circovirus diseases. Moreover, our finding also raised an interesting question about whether enhancing the levels of Rep and Rep’ protein in cells facilitates the virus replication of PCV2. In addition, our study revealed that the genome copy number and TCID_50_ of the two parental virus strains YiY-3-2-1 and YiY-3-2-10 are significantly different. We speculated that the difference might be derived from the alterations of two amino acids at the N-terminal of the ORF1 between them, or this might be associated with the difference in the Cap proteins of them. Notably, compared with that of YiY-3-2-1 strain, there was an amino acid (lysine) elongation at the C-terminus of YiY-3-2-10 strain [43]. Further investigations are required to clarify these.

## Conclusion

In summary, this study for the first time demonstrates that the infectious clones which have 1.1 copy of PCV2 without or with point mutations in the ORF1 can be recovered in vitro and the naturally occurring G^710^ to A^710^ mutation resulting in C-terminal truncated Rep protein reduces viral replication and infectivity. The information reported here facilitates future investigations on the contribution of the ORF1 to the virulence and pathogenicity of PCV2.

## Data Availability

Raw data for tables and figures of the current study are available from the corresponding authors on reasonable request.

## Abbreviations

Cap: Capsid
CT: Congenital tremors type A-II
DMEM: Dulbecco’s Modified Eagle Medium
FBS: Fetal bovine serum
FITC: Fluorescein isothiocyanate
IFA: Indirect immunofluorescence assay
IgG: Immunoglobulin G
NC: Negative control
ORFs: Open reading frames
PBS: Phosphate buffered saline
PC: Positive control
PCR: Polymerase chain reaction
PCV2: Porcine circovirus 2
PCVADs: Porcine circovirus-associated diseases
PDNS: Porcine dermatitis and nephropathy syndrome
PK15: Porcine kidney cell line
PMWS: Postweaning multisystemic wasting syndrome
PRDC: Porcine respiratory disease complex
TCID_50_: Tissue culture infective dose 50
TEM: Transmission electron microscopy
VLP: Virus-like particle
